# Insecticidal efficacy of residual spraying with deltamethrin–clothianidin (Fludora^®^ Fusion) in Papua New Guinea

**DOI:** 10.1186/s13071-026-07310-7

**Published:** 2026-02-22

**Authors:** Evodia Anetul, Petrina Johnson, Rebecca Vinit, Nakei Bubun, Kiari Kiari, Rowena Absalom, Daniel Aulim, Paul Daly, Melanie Koinari, Tiziano Raffaelli, Jason H. Richardson, Michael Macdonald, Jacob Kisomb, Maria Ome-Kaius, Moses Laman, Leanne J. Robinson, Stephan Karl

**Affiliations:** 1 Vector-Borne Diseases Unit, Papua New Guinea Institute of Medical Research, Madang, Papua New Guinea; 2 Australian Institute of Tropical Health and Medicine, James Cook University, Smithfield, QLD Australia; 3 Rotarians Against Malaria Papua New Guinea, National Capital District, Port Moresby, Papua New Guinea; 4 Burnet Institute, Melbourne, VIC Australia; 5 Innovative Vector Control Consortium, Liverpool, UK; 6 Papua New Guinea National Department of Health, National Capital District, Port Moresby, Papua New Guinea

**Keywords:** Fludora^®^ Fusion, Residual spraying, *Anopheles farauti*, Efficacy, Wall surfaces

## Abstract

**Background:**

In Papua New Guinea (PNG), insecticide-treated nets leave considerable gaps in protection from *Anopheles* mosquito bites and, as a result, malaria cases are increasing. Residual spraying (RS) may be an effective complementary malaria control strategy. To understand the potential impact of RS and guide RS implementation, it is important to quantify the duration of insecticidal efficacy of RS formulations. The materials used to construct houses in rural PNG differ significantly from the African study sites where most such data have been generated. This study investigated determinants of duration of insecticidal efficacy of Fludora^®^ Fusion, a co-formulation of clothianidin and deltamethrin in two villages in PNG, following a RS intervention study.

**Methods:**

Eight sprayed houses and four control houses with different wall materials (six sago palm, six bamboo) were selected from each village. Test surfaces were either inside the houses or in protected outdoor locations such as on verandas, which had also been sprayed. Mortality at 24 h post exposure (M24h) was measured over 11 months post RS with wall cone bioassays and susceptible *Anopheles farauti* colony mosquitoes. The duration that M24h remained above 80% was estimated using a statistical model.

**Results:**

M24h exceeded 95% on all sprayed surfaces within 1 month after spraying but decreased by 51 percentage points during the study period. Overall, M24h fell below 80% at 25 weeks post RS but variability was high [95% confidence interval (CI) 9–41 weeks]. The duration of insecticidal efficacy was significantly associated with the application rate, surface material, indoor versus outdoor location and height of the tested surface area.

**Conclusions:**

The duration of insecticidal efficacy of Fludora^®^ Fusion (M24h > 80%) was an average of 6 months in these field conditions but was highly variable and depended significantly on surface parameters. RS with Fludora^®^ Fusion could be a valuable addition to insecticide-treated nets (ITNs) to control malaria in PNG. However, frequent reapplication may be required depending on surface properties and location. Achieving consistent target application rates is critical to achieve maximum duration of insecticidal efficacy.

**Graphical abstract:**

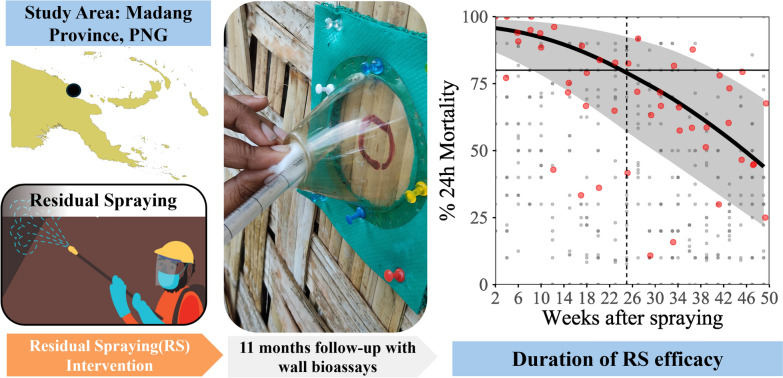

**Supplementary Information:**

The online version contains supplementary material available at 10.1186/s13071-026-07310-7.

## Background

Residual spraying (RS) is used as a vector control intervention in malaria endemic countries [1]. The method works by applying long-lasting insecticide formulations, usually to indoor surfaces of houses (‘indoor RS’ or IRS). While RS predates insecticide-treated nets (ITNs) and was a main vector control intervention during the global malaria eradication campaign, current coverage with RS is much lower than that with ITNs [2, 3]. Although the epidemiological impact of RS is not as well studied as that of ITNs [4], there is considerable evidence that RS significantly reduces malaria incidence, reviewed, for example, by Zhou Y et al. [5]. It is also well established that RS provides significant additional benefit when co-deployed with ITNs [6, 7].

Papua New Guinea (PNG) and neighbouring Solomon Islands have the highest malaria incidence outside of sub-Saharan Africa [8]. Malaria incidence in coastal and lowland regions of PNG are comparable with the highest burden African scenarios, with the malaria-free but densely populated PNG highlands contributing to lower average national incidence rates [9]. ITN mass distributions have helped reduce average reported national malaria incidence in PNG from > 200 per 1000 per year before 2009 to < 50 per person per year in 2015 [10]. Since 2015, average reported national malaria incidence in PNG has been rising again owing to various potential contributing factors, including reduced community protection from the distribution of inferior ITN products [11], commodity shortages and changes in human behaviour resulting in increased human-to-vector contact [12, 13]. Generally, there is a consensus that ITNs cannot interrupt all transmission even if universal coverage with highly efficacious products is achieved, as they leave ‘gaps’ in the protection of individuals [14]. These gaps include times when people, even though they may have access to ITNs, are not protected by them, such as the early evening and morning hours. Previous studies have shown that outdoor human–vector contact in PNG is high [15]. Some evidence also suggests low or decreasing ITN coverage and usage in PNG [16, 17]. Nationally, estimated ITN access is 57.4% and usage is 43.6% [18]. However, ITN coverage and usage rates can vary by location and source, and in Madang province, where this study was conducted, 85% of the population have access to ITNs and usage is estimated to be 78% [18] yet malaria rates remain high.

Additional vector control strategies should therefore be explored to complement ITN distributions, and one such strategy is RS. Given that in PNG the majority of malaria transmission occurs in the rural peri-domestic setting and is facilitated by vectors that bite early in the evening and outdoors [19], RS targeting indoor and especially protected outdoor surfaces is a potentially effective additional vector control method that may be useful in high burden case reduction and focal vector control scenarios.

An indoor RS program with dichlorodiphenyltrichloroethane (DDT) was implemented in PNG between 1957 and the early 1980s, before being discontinued owing to operational constraints. The program was extensive, involving several thousand spray operators and covering approximately 50% of the country. Surveillance reports from the time suggest that malaria epidemics ceased in the highlands and parasite rates were substantially reduced in other areas [20]. To inform potential re-introduction in PNG, a feasibility-focused RS study was conducted in two villages in Madang Province, PNG. The insecticide used was Fludora^®^ Fusion (Bayer AG), a co-formulation combining clothianidin (neonicotinoid) and deltamethrin (pyrethroid). Fludora^®^ Fusion was pre-qualified by the World Health Organization (WHO) for use in IRS in 2018 [21]. The addition of clothianidin presents a new mode of action which offers a prospect to alleviate the emergence and spread of insecticide resistance in malaria vectors, making it relevant for the PNG setting, where all *Anopheles* populations remain pyrethroid susceptible [22, 23].

In the present study, the duration of efficacy of Fludora^®^ Fusion to kill pyrethroid susceptible *Anopheles farauti* s.s. colony mosquitoes was assessed, *An. farauti* being one of the major malaria vectors in PNG [24]. Duration of insecticidal efficacy of RS may differ with varying spray quality, wall surface materials and location of walls indoors or outdoors. Limited data exist for the duration of insecticidal efficacy on surfaces commonly found in traditional houses in PNG, such as walls constructed from plant-based materials including bamboo and sago palm, as well as for protected outdoor surfaces, which may be relevant spray targets in the PNG setting due to the high proportion of outdoor biting [25]. In addition, there is a scarcity of data describing the impact of RS against local vectors, such as *An. farauti*.

The present study represents a substantial contribution to knowledge on the expected performance of RS in PNG settings. The data presented in this study can be generalized to some extent to a range of malaria-endemic Pacific Island settings with similar *Anopheles* species, malaria transmission environments and house types [26, 27].

## Methods

### Study area and residual spray application

**Fig. 1:**
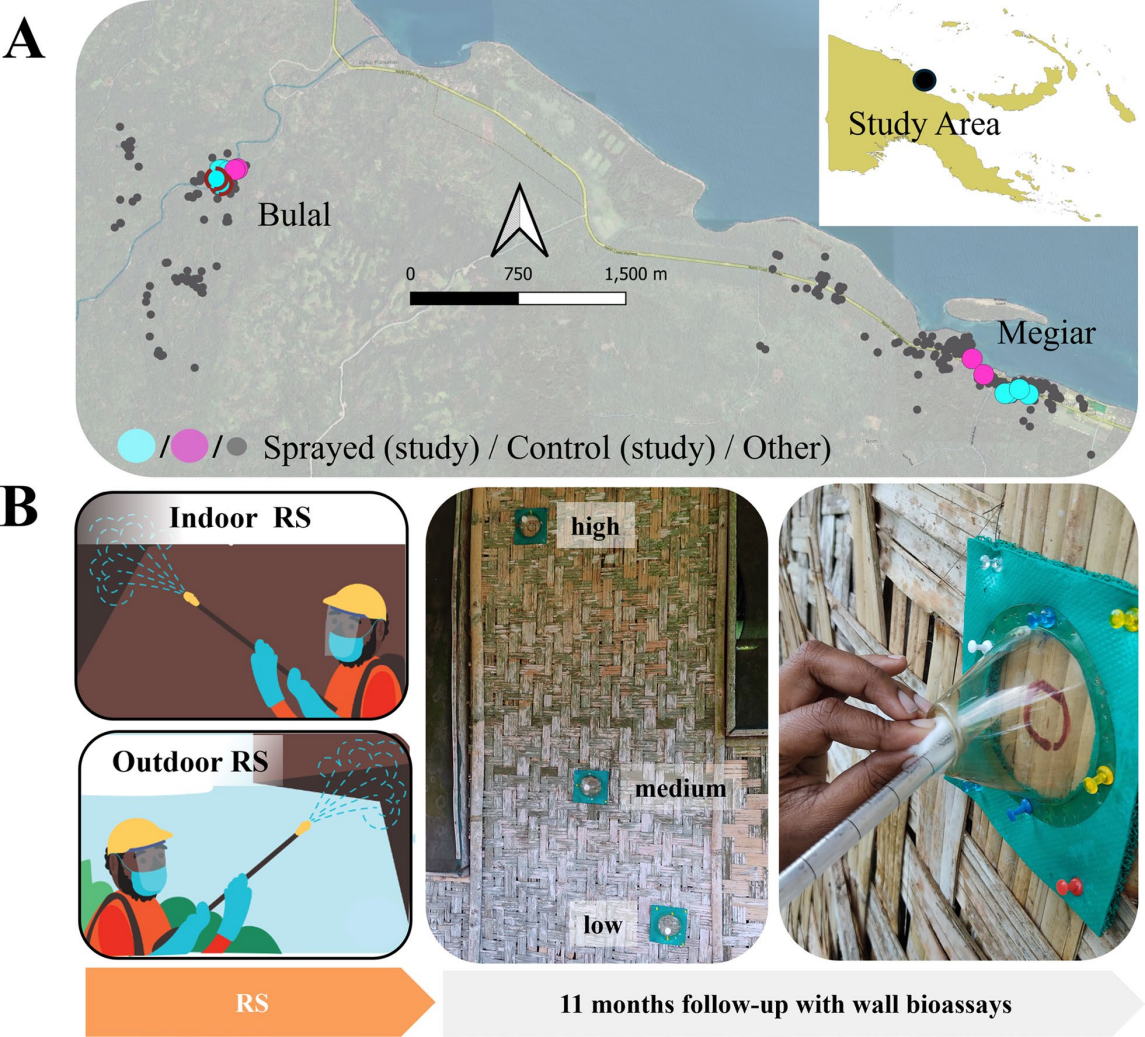
Study area and design. Panel **A** The study was conducted in two villages, Megiar and Bulal, in Madang Province in PNG. The map shows the villages and study houses, and the inset shows where in PNG the study area is located. Panel **B** Residual spraying with Fludora^®^ Fusion was implemented in the two villages in November 2021 on indoor and protected outdoor surfaces. Wall cone bioassays were conducted on sprayed surfaces (*n* = 8 houses) and control surfaces (*n* = 4 houses) in regular intervals for 1–11 months after RS application. One study house was changed to another after 6 months owing to owner withdrawal of consent (indicated as cyan circles with a red border in the map)

The study was conducted in two villages, Megiar and Bulal, in the Sumkar District of Madang province in PNG (Fig. 1A). The villages were part of a RS feasibility study in which > 80% of houses were sprayed with Fludora^®^ Fusion in November 2021.

Megiar is situated directly on the coast, whereas Bulal is located ~2 km inland from the coast (Fig. 1A). The coastal area (Megiar) is characterized by coconut and cocoa plantations, gardens worked for subsistence farming and river mouths surrounded by saline swamps. Inland (Bulal), the landscape consists of steep sided hills covered with tropical rain forest and gardens for subsistence agriculture. The average monthly rainfall in the study area is 250–350 mm. The wet season is from October to May and the dry season is from June to September.

There was a total of 330 households in the two villages, most of them traditional houses. The walls in the majority of houses in the study villages are made from ‘bush materials’, bamboo (49.2%) and sago (35.4%). Other, less commonly used building materials include metal sheet (6.4%), masonite/fibre cement (6.3%), timber (1.7%), plywood (0.6%), cement/bricks (0.2%) and others (not further defined, 0.4%) (Supplementary Fig. S1).

RS was applied according to WHO guidelines [28] by trained technicians in consultation with village leaders, the Provincial Health Authority (PHA) of Madang Province and the PNG National Malaria Control Program. Each main house and its sub-structures’ surfaces were sprayed including all accessible indoor surfaces and protected outdoor surfaces. Technical spray personnel were supervised closely to ensure that the application was conducted as per protocol and guidelines, with a target dose of 25 mg/m^2^ deltamethrin and 200 mg/m^2^ clothianidin.

### Household selection and study design

Houses for the present study were purposively selected from the census database created prior to the study. Four sprayed houses were selected from each village, which included two houses with bamboo walls and two houses with sago palm walls, for a total of *n* = 8 sprayed houses. Two unsprayed houses from each village with the same wall type were selected as controls, so that the total number of study houses across the two villages was *n* = 12. Verbal consent was obtained from the heads of the households before conducting wall cone bioassays. Wall cone bioassay surveys were conducted following a set schedule, with the first bioassay at 2 weeks (Bulal) or 4 weeks (Megiar) after spraying followed by ~4-weekly repetitions for 11 months (up to 346 days after spraying).

### Mosquito rearing and transportation

Pyrethroid-susceptible *An. farauti* colony mosquitoes were used in the wall cone bioassays. Test mosquitoes were reared in the insectary at the PNG Institute of Medical Research insectary in Madang, approximately 2 h (44 km) drive away from the study villages. This necessitated transportation of live mosquitoes to the study sites and presented a considerable logistical challenge. Using mouth aspirators, 2–4-day old mosquitoes were transferred from cages into paper cups screened with untreated netting. Pads made from cotton wool soaked in 10% sugar solution were placed onto each cup. The holding cups were labelled and placed into cooler boxes. Plexiglass sheets were placed over the holding cups and two frozen ice bricks wrapped in dry tea towels were placed on top the plexiglass sheets to maintain cool, low moisture conditions during transport. At the field site, test mosquitoes were held overnight before conducting the wall cone bioassays. The percentage of test mosquitoes that survived transportation throughout study was > 95%. Temperature and humidity loggers were placed into the cooler boxes for monitoring conditions.

### WHO wall cone bioassays

The wall cone bioassays were carried out using standard methods, with small modifications to adapt the method to local settings, as follows [29].

The bush material walls of PNG village houses often exhibit uneven surfaces, gaps and crevices allowing potential escape routes for mosquitoes during the exposure in the assays. To minimize this, and to be able to fit the cones firmly to the wall surfaces, flexible rubber spacers were cut out to fit the diameter of the cones (Fig. 1B). The rubber spacers were attached to the walls using push pins, effectively sealing any gaps between the cone and the walls (Fig. 1B). Any further gaps in the walls underneath the cones were filled with cotton to prevent mosquitoes escaping and a board was placed behind the wall to reduce light intensity during the test.

In each of the selected houses, one wall surface indoors and one wall surface in an outdoor sheltered area were selected. Three WHO bioassay cones (Fig. 1B) were attached to each wall, at three different heights: low, medium and high (Fig. 1B). The position heights from floor level were approximately 20–30 cm (low), 110–120 cm (medium) and 195–200 cm (high), depending on the wall height in each household and the field team’s safe reach limit. The bioassay test position was marked on the wall and the same position was tested at each time point, with care being taken to avoid touching the test surface.

Ten unfed, female *An. farauti* mosquitoes were introduced into the cones using mouth aspirators. After 30 min of exposure to the wall surfaces, the mosquitoes were transferred into screened plastic containers with access to 10% sugar solution on a cotton wool pad. The percentage knock down after 30 min of exposure (KD30m), 24-h mortality (M24h), that is, the proportion of dead mosquitoes after a 24 h holding period, and 48 h mortality (M48h) were recorded. All mosquitoes that could fly were considered alive.

Similar control experiments were conducted on the same day, using separate equipment. Test mortality was not adjusted with the observed control mortality as these experiments were conducted in different houses, and thus cannot be considered paired. When control mortality was greater than 20% on average, all tests with mosquitoes from the same batch were either repeated or excluded from further analysis.

### Target dose verification with filter paper samples

Spray application on wall surfaces was assessed by measuring insecticide residue on filter paper samples attached to walls in the study houses. Filter papers (Whatman^®^ No. 1) were attached to wall surfaces in positions immediately adjacent to wall cone bioassay positions as described above. Each paper was attached to a square of aluminium foil with adhesive putty so that the paper was offset from the foil, and the foil was then pinned to the wall surface. Filter papers were set up on the morning of spraying and removed when papers were dry, approximately 24 h later. Filter papers were wrapped in aluminium foil and labelled with date, household code and position details and then stored at approximately 4 °C until chemical analysis using high-performance liquid chromatography (HPLC).

### High-performance liquid chromatography (HPLC)

Deltamethrin was extracted from 2 cm × 2 cm filter paper samples and analysed following previously described methods [30]. For clothianidin, each 2 cm × 2 cm filter paper sample was submerged in 400 µL of acetonitrile (≥ 99.9%, gradient grade, suitable for HPLC; Merck, Australia) and left at room temperature for 10 min before adding 1600 µL of distilled water (acetonitrile/dH₂O, 20:80, v/v). This solvent composition minimized early peak elution (at approximately 2 min) during HPLC analysis. HPLC analysis was performed using an Agilent 1260 Infinity system equipped with a C18 preparative column (Phenomenex Jupiter 10 µm, C18, 300 Å, 250 mm × 21.2 mm; Phenomenex) operating at a flow rate of 1 mL/min. The mobile phase consisted of two components: (solvent A) 0.05% trifluoroacetic acid (TFA) (≥ 99.9% purity; Chem-Supply, Australia) in water and (solvent B) 65% acetonitrile, 35% water and 0.1% TFA. Samples (20 µL) were eluted isocratically in 25% solvent B over 12 min, and UV absorbance was monitored at 254 and 280 nm. Analytical-grade standards of deltamethrin and clothianidin were purchased from Merck (Australia) for quantification. HPLC measurements were converted to mg/m^2^ via the standard curve and the known weights of the measured samples.

### Data analysis

M24h was considered the primary outcome measure. KD30m was considered a secondary outcome measure as it is less robust, especially when results are interpreted in the field. M48h was not used in the models owing to high control mortality.

Binomial mixed-effects generalized models (GLMMs) were used to explore associations between the properties of the sprayed walls and the observed insecticidal efficacy (i.e. M24h and KD30m). The properties considered in the models were (i) the time after spraying, (ii) inside or outside wall location (factor variable), (iii) bamboo versus sago wall material (factor variable), (iv) insecticide concentration at baseline (log transformed to account for dose–response behaviour) and (v) the position on the wall where the bioassays were conducted (factor variable, low, medium or high on the wall). The model structure accounted for clustering of repeated observations within households and on separate surfaces within households using a nested correlation structure.

The GLMM was run overall and also separately for indoor versus outdoor, wall material and wall height factors to graphically explore the impact of these different properties on M24h and KD30m.

The duration that M24h remained above 80% was estimated using the model predictions, with M24 > 80% being the WHO-recommended threshold for acceptable activity of RS applications [31].

## Results

**Fig. 2:**
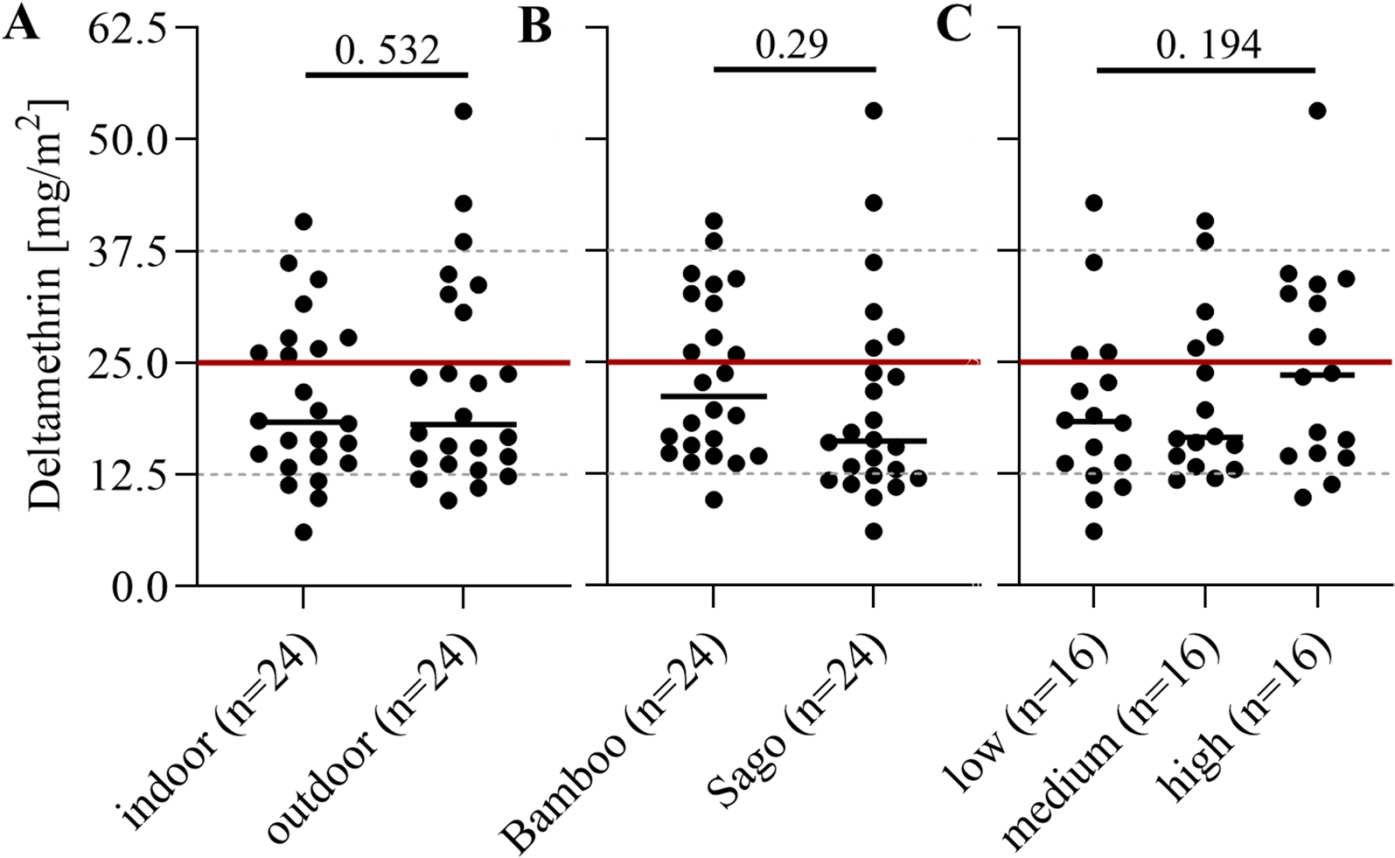
Deltamethrin concentrations on the sprayed surfaces subject to wall bioassays. Panel **A** Indoor versus outdoor surfaces, Panel **B** Bamboo walls versus sago walls, Panel **C** Low, medium and high areas on the walls. The horizontal lines are the application target range of deltamethrin (25 mg/m^2^ ± 50%)

The chemical analysis of the surfaces at baseline indicated that 83% (40/48) of the tested surfaces had received at least the lower limit of the target dose of 12.5 mg/m^2^ deltamethrin as shown in Fig. 2. It is assumed that deltamethrin and clothianidin are a homogenous mixture and, therefore, that deltamethrin concentration is an appropriate proxy for overall target dose of both active ingredients. This is supported by the strong correlation between deltamethrin and clothianidin HPLC measurements (*R*^2^ = 0.82, *P* < 0.001, also refer to Supplementary Fig. S2).

There was no statistically significant difference in insecticide concentration on indoor versus outdoor surfaces (Fig. 2A, *t*-test, *P* = 0.52). There was also no statistically significant difference between surfaces made from bamboo versus sago (Fig. 2B, *P* = 0.29), but one of the sago houses had received lower average doses on most surfaces, such that four out of the six test surfaces in this house were under-sprayed with less than 50% of the target dose. As such, 50% of all under-sprayed surfaces in the study were concentrated in one house. A sub-analysis was conducted on the data excluding this house to confirm that the model estimates remained consistent.

While there was no statistically significant difference in the insecticide concentration on the lower areas of the walls as compared with the higher areas of the walls (Fig. 2C, *P* = 0.19), there was an overall trend towards higher concentrations on higher wall areas, potentially indicating a bias in the spray application. These observations may be relevant when interpreting the results of the multivariate model predicting the duration of efficacy of Fludora^®^ Fusion on these various surfaces.

**Fig. 3:**
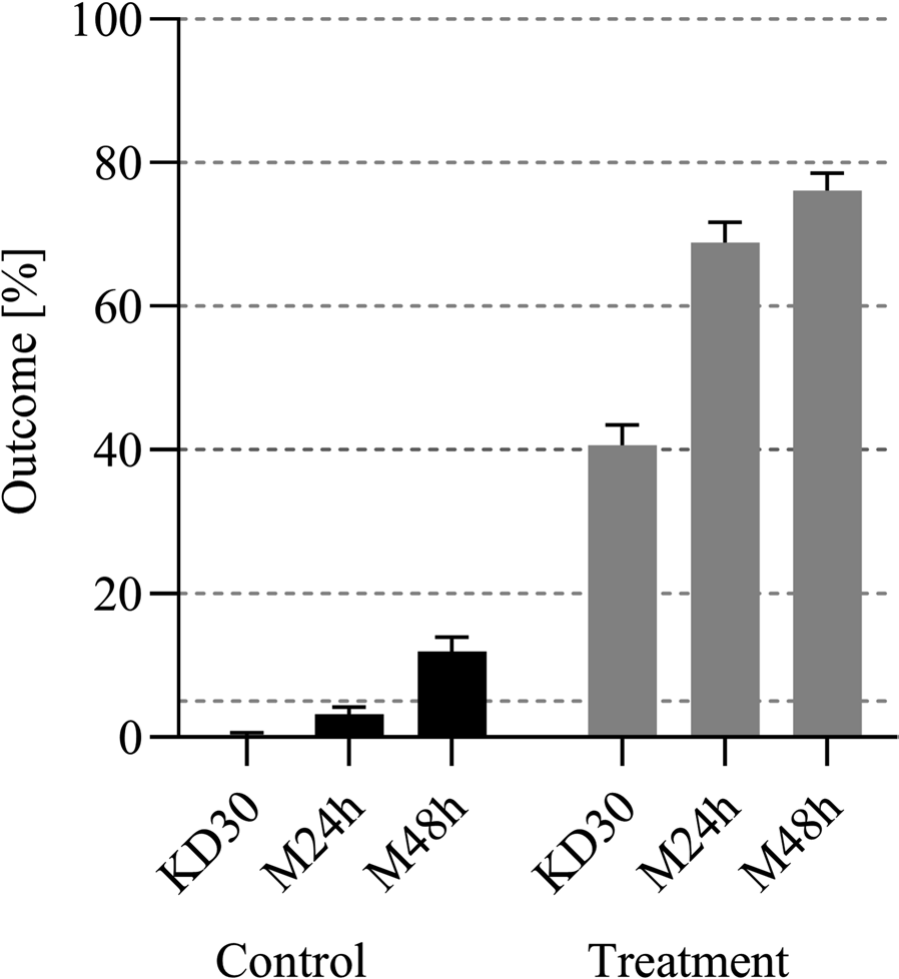
Overall comparison of 30-min knockdown (KD30), 24-h mortality (M24h) and 48-h mortality (M48h) outcomes for control and treatment surfaces. There was no evidence of delayed mortality

Overall 24-h mortality on sprayed walls was 69%, while 48-h mortality was 76% (Fig. 3). At the same time, overall control M24h was < 5% while overall control M48h was 12%. Given that test and control mortality increased by a similar amount between 24 and 48 h, there was no evidence for delayed mortality.

**Fig. 4:**
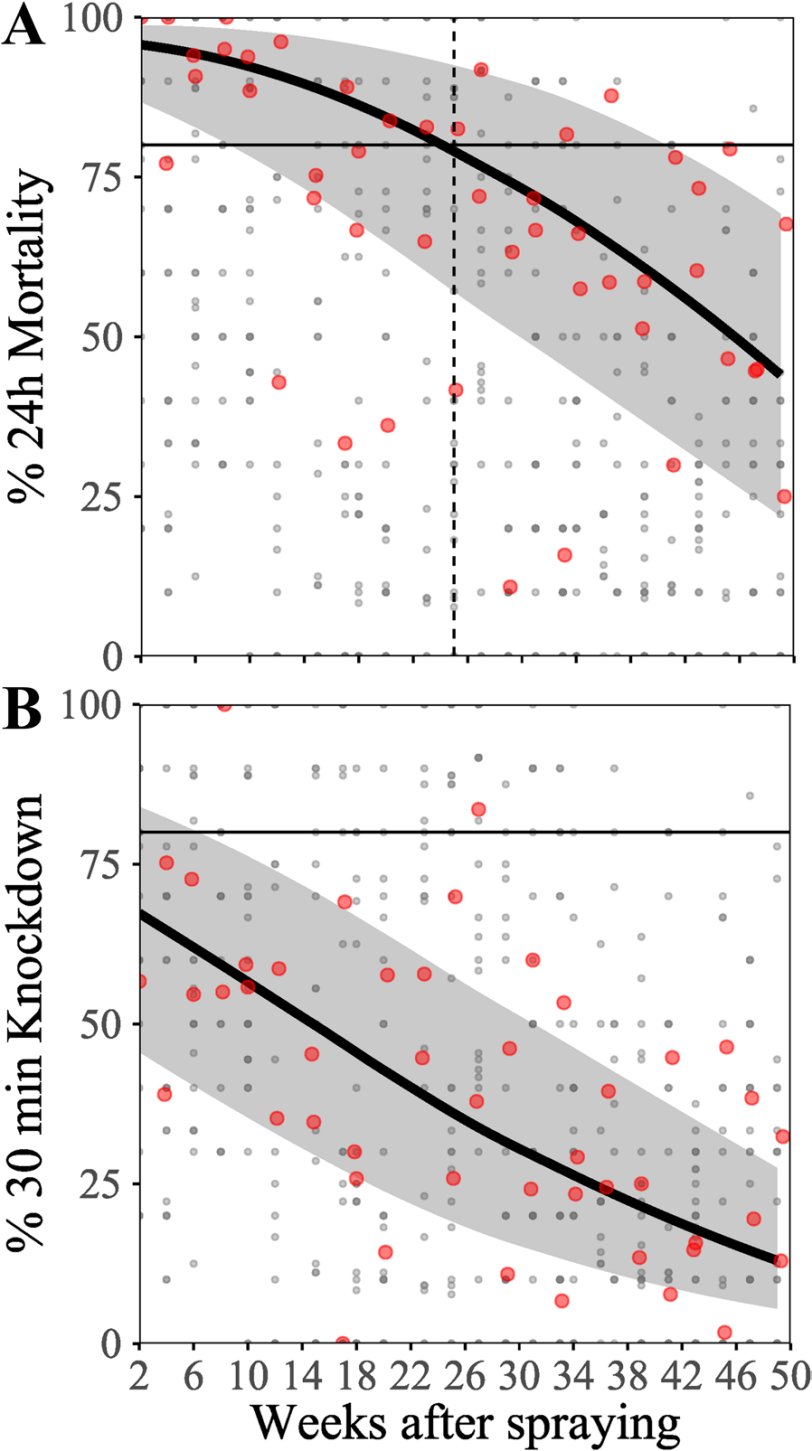
Overall association of 24-h mortality (M24h) and 30-min knockdown (KD30m) with time after spraying. Panel **A** 24-h mortality/time after treatment; Panel **B** 30 min knock down/time after treatment; The continuous lines and bands in each the average predictions and the 95% confidence bands, smoothened with locally estimated scatterplot smoothing (LOESS). The red circles are the averages of all bioassays conducted at a particular follow-up time point. The small grey dots are the individual assay raw data. In Panel **A**, the dashed horizontal line denotes 80% M24h and the vertical dashed line denotes the time when the average model estimate intersects with the 80% M24h line, that is, the estimated duration of efficacy. Time is presented in exact days and not rounded to weeks

While the primary outcome, M24h, was close to 100% (> 95%) on all surfaces 2–4 weeks after spray application, insecticidal efficacy gradually decreased over time (Fig. 4A). The model estimated an average decrease by about 51 percentage points during the 333 (i.e. from day 13 to 346 after spraying) day measurement period (Fig. 4A). The average predicted M24h fell below 80% at 25 weeks [95% confidence interval (CI) 9–42 weeks] after spray application but remained over 50% for most of the follow-up period.

The secondary outcome, KD30m, was statistically significantly, but loosely correlated with M24h with a Spearman *R* of 0.65, *P* < 0.001 (Spearman Rank correlation). KD30m was systematically lower as compared with M24h and decreased by about 54 percentage points during the study period when the same model as that used for M24h was applied.

M24h was also significantly associated with the deltamethrin content measured at baseline [2.82 (1.28–6.18)] per log increase in deltamethrin, *P* < 0.010, Table 1, Supplementary Fig. S4).

**Table 1:** Coefficients of the generalized linear mixed-effects model of percentage 24-h mortality and 30-min knockdown

Effect	OR (95% CI)	*P*
24-h mortality (M24h) model		
Indoor (ref: outdoor)	2.78 (1.59–4.86)	0.003
Sago walls (ref: Bamboo walls)	0.17 (0.03–0.95)	0.044
Wall position (ordinal, per level, ref: high)	0.49 (0.29–0.80)	0.005
Log deltamethrin concentration (mg/m^2^)	2.82 (1.28–6.18)	0.010
Time (per week)	0.92 (0.90–0.93)	< 0.001
30-min knockdown (KD30m) model		
Indoor (ref: outdoor)	1.64 (1.13–2.39)	0.009
Sago walls (ref: bamboo walls)	0.40 (0.11–1.44)	0.160
Wall position (ordinal, per level, ref: high)	0.59 (0.42–0.82)	0.002
Log deltamethrin concentration (per mg/m^2^)	2.21 (1.30–3.77)	0.003
Time (per week)	0.94 (0.93–0.95)	< 0.001

Figure 4 shows the overall average insecticidal efficacy over time after spraying, in terms of M24h and KD30m.

**Fig. 5:**
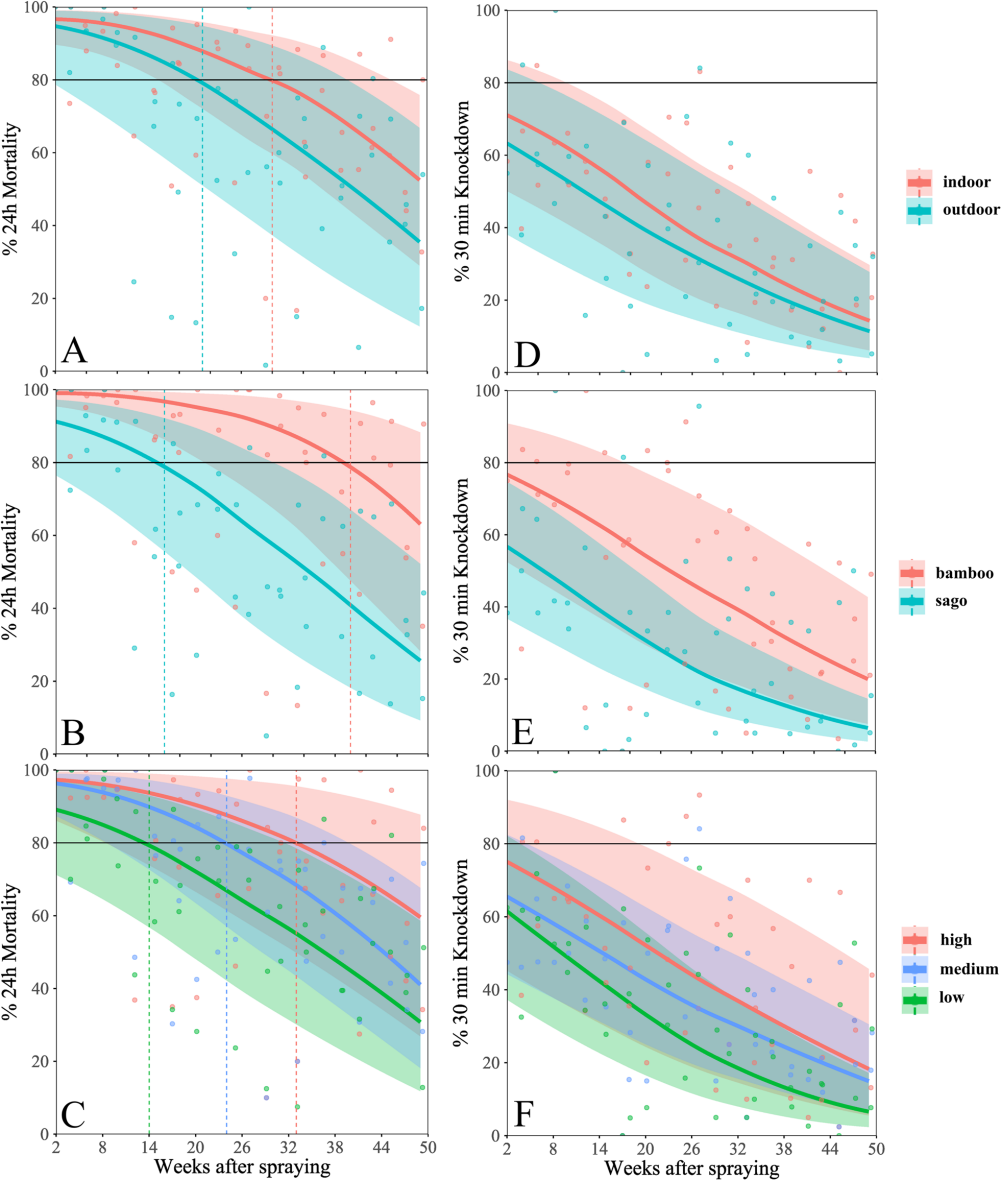
Association of wall properties, time after spraying and 24h mortality (M24h) and 30-min knockdown (KD30m). Panels **A**, **B** and **C** are for M24h and panels **D**, **E** and **F** are for KD30m. Panels **A** and **D** Wall location, indoor or outdoor; Panels **B** and **E** Wall material: bamboo versus sago palm; Panels **C** and **F** Position of the bioassay cone on the wall: high, medium, low. Continuous lines are the population-level predictions and the 95% confidence bands (shaded areas), smoothened with locally estimated scatterplot smoothing (LOESS). The circles represent the averages of all values measured on surfaces with the specific property at a follow-up time point. In panels **A**, **B** and **C**, the dashed horizontal line denotes 80% M24h and the coloured vertical dashed lines denote at which time the average model estimate intersected with the 80% M24h line

Apart from time after spraying and deltamethrin concentration, wall properties were also significantly associated with M24h. M24h was significantly higher on indoor surfaces as compared with outdoor surfaces [odds ratio (OR) 2.78, *P* < 0.003, Table 1, Fig. 5A], resulting in an average 16 percentage point lower M24h on outdoor surfaces at the end of the study. Potentially, outdoor walls may have been more frequently exposed to conditions detrimental to the duration of efficacy of the product. This may include, for example, increased or partial exposure to UV light, precipitation and abrasion.

M24h was significantly lower on sago walls compared with bamboo walls (OR 0.17, *P* < 0.044, Table 1, Fig. 5B), such that sago walls had 50 percentage points less M24h at the end of the study as compared with bamboo walls but with a wide confidence interval. This may be related to different absorption properties of these materials.

The height at which wall bioassays were conducted was also associated with M24h. Medium and lower height wall areas showed a statistically significantly lower M24h with OR 0.49 per level (*P* = 0.005, Table 1, Fig. 5C). This may be related to systematic inconsistencies in spray technique, such that lower parts of the walls had received less insecticide (see Fig. 2C) or increased exposure of lower wall sections to conditions detrimental to the duration of efficacy of the product, such as abrasion, as people or domestic animals may touch them more frequently.

Associations with KD30m generally followed the same trend as those observed with M24h (Table 1).

Results from the separate models for the different wall properties are shown in Fig. 5. Wall properties had an impact on the estimated duration of M24h to remain above 80%. For example, outdoor surfaces were predicted to fall below 80% M24h at around 21 weeks (95% CI 0–40 weeks) post spraying while the effect on indoor surfaces lasted for 31 weeks (95% CI 15–46 weeks). M24h of bamboo walls remained above 80% for an average of 41 weeks (95% CI 22 to > 50 weeks), while M24h on sago walls fell below 80% after 17 weeks (95% CI 0–30 weeks). Higher wall areas lasted longer, with 33 weeks (95% CI 9 to > 50 weeks) > 80% as compared with lower wall areas, with 15 weeks (95% CI 0–32 weeks). In general, KD30m followed the same trends as M24h.

Notwithstanding these differences, the overall average M24h remained above 80% for 6 months for the entire 346 days after spray application, indicating the potential value of RS in PNG rural settings.

## Discussion

This study assessed the duration of insecticidal efficacy of Fludora^®^ Fusion, a clothianidin and deltamethrin mixture for RS with pyrethroid-susceptible *An. farauti* colony mosquitoes in PNG, under field conditions. All wild *Anopheles* populations in PNG are currently still indicated to be pyrethroid-susceptible [32] and, as such, these results can be considered as indicative of the response that could be expected from wild *Anopheles* populations in this scenario. It may be relevant to consider a non-pyrethroid RS formulation in this setting to delay the emergence of pyrethroid resistance and preserve the efficacy of current ITNs [33, 34].

Overall, average M24h was observed to remain above 80% for 25 weeks post application, regardless of surface material or wall location. This seems relatively short, given the susceptibility status of the mosquitoes. For all indoor surfaces, average M24h remained above 80% for 31 weeks (7 months).

It is difficult to compare these findings with those from previous studies, as studies on surfaces other than cement or mud, and from non-African settings, are rare. Studies conducted with Fludora^®^ Fusion and susceptible *An. gambiae* (Kisumu) mosquitoes have reported > 80% M24h for over 9 months indoors on treated cement surfaces, but some studies found lower durations of efficacy for mud surfaces (3 months), similar to the indoor surface results in the present study and highlighting the potential importance of the surface material [35–37].

The present study thus shows that it is critical to consider surface material and location and also the position of the bioassay cones in the interpretation of duration of efficacy results in field studies, where these parameters may be less controllable as compared with laboratory or semi-field studies. Bush material walls rarely present uniform surfaces, and natural gaps and undulations are expected to result in inconsistent deposition of insecticide spray droplets. Further biochemical and biophysical studies may elucidate surface properties (e.g. related to absorption), that are beneficial or detrimental to the duration of insecticidal efficacy of RS products [38]. The results indicate that spray application rate varied considerably and this may somewhat limit the interpretation of residual efficacy bioassay results, in particular given the low sample size.

Duration of insecticidal efficacy of Fludora^®^ Fusion may be shorter against pyrethroid resistant populations (e.g. 4 months) [37]. However, studies from India and Africa also indicated that Fludora^®^ Fusion had 6–12 months duration of efficacy (M24h > 80%) against pyrethroid resistant mosquitoes indoors, on mud and cement surfaces [37, 39].

Clothianidin is known to elicit delayed mortality with pyrethroid resistant mosquitoes and several previous laboratory and experimental hut studies thus measured mortality at 96 h or 120 h post exposure [36, 40]. Clothianidin-induced delayed mortality can be less pronounced in pyrethroid susceptible populations [41] and is not considered relevant when clothianidin is used in combination with deltamethrin and susceptible mosquitoes as in this study. There was no evidence for delayed mortality with the susceptible *An. farauti* colony mosquitoes in this study. M48h was on average increased by 7% (95% CI 2–13%) over M24h. At the same time, control mortality increased by 8% (95% CI 6–12%), indicating that the added mortality at 48 h was not due to exposure to insecticide (Fig. 3). However, 30 min was clearly too short for the exposure to unfold the full effect of the insecticide as indicated by the substantially and systematically lower KD30m outcomes as compared with M24h (Fig. 4).

Limitations of this study include a small sample size of *n* = 8 treatment houses. Clustering of the repeated measures within houses was significant but accounted for in the statistical model. In addition, spray application was observed to be variable despite the fact that the spray operators were closely supervised. Other studies have shown high variability of insecticide surface concentration after spray application due to spray operator technique [42, 43]. Thus, variation in insecticide concentration on the test surfaces can be expected to influence the present results. One house made from Sago had received a lower average insecticide concentration (Supplementary Fig. S3), and overall a higher proportion of sago surfaces (7/24 versus 1/24 bamboo) had received a dose of Fludora^®^ Fusion that was less than 50% of the target dose. However, the effect of surface material remained significant when the under-sprayed surfaces (< 12.5 mg/m^2^) were excluded from the analysis, with OR 0.26 (0.23–0.30), *P* < 0.001.

The field site was a 2 h drive from the insectary, necessitating colony mosquito transport by car and acclimatization of the colony mosquitoes at the field site over night before the assays could be conducted. Despite substantial effort into method development and the utmost of care being taken, this sometimes resulted in higher than desirable control mortalities, especially at longer holding times (M48h). However, it is unlikely that the results of the present study were impacted significantly by this, as 24 h control mortalities were still generally low (average < 5%). The remoteness of the study site also meant that sometimes tests were delayed and the monthly schedule could not be strictly adhered to. The statistical models explicitly used ‘days after spray application’ and thus accounted for these variations in the follow-up time.

Despite these limitations the overarching trends observed in this study are clear and provide some guidance for RS implementation in rural PNG settings. Further research quantifying *Anopheles* indoor resting behaviours in PNG would be useful to further contextualize the present findings.

## Conclusions

The results of this study show that in practice, RS with Fludora^®^ Fusion in PNG rural settings can exhibit variable duration of efficacy, with an overall 95% CI range of 9–41 weeks of M24h > 80% (average 25 weeks). The main determining factors identified through this study were application rate, wall location and indoor versus outdoor, with outdoor surfaces prone to a faster decrease of insecticidal efficacy. Wall material played a significant role, with bamboo resulting in longer duration of insecticidal efficacy. More studies are needed on traditional housing materials in PNG and similar settings. The study confirms that RS with Fludora^®^ Fusion in PNG villages with predominantly ‘bush material’ houses, on average, can provide protection long enough to cover the peak malaria transmission months in a PNG setting (October to May). However, given that malaria transmission in PNG is perennial, annual or even more frequent, re-application would have to be considered if the goal was to achieve year-round efficacy of RS.

## Supplementary Information

Supplementary material 1.

## Data Availability

Data supporting the main conclusions of this study are included in the manuscript.
