# Supplementary material for: Insecticidal efficacy of residual spraying with deltamethrin–clothianidin (Fludora® Fusion) in Papua New Guinea
Source: Parasit Vectors. 2026 Feb 22;19:136. doi: 10.1186/s13071-026-07310-7 (PMC13032584; doi:10.1186/s13071-026-07310-7)
Supplement: Supplementary file 1 — Supplementary material 1. [file 13071_2026_7310_MOESM1_ESM.docx]

# **Supplementary Information**

**Additional Figures**


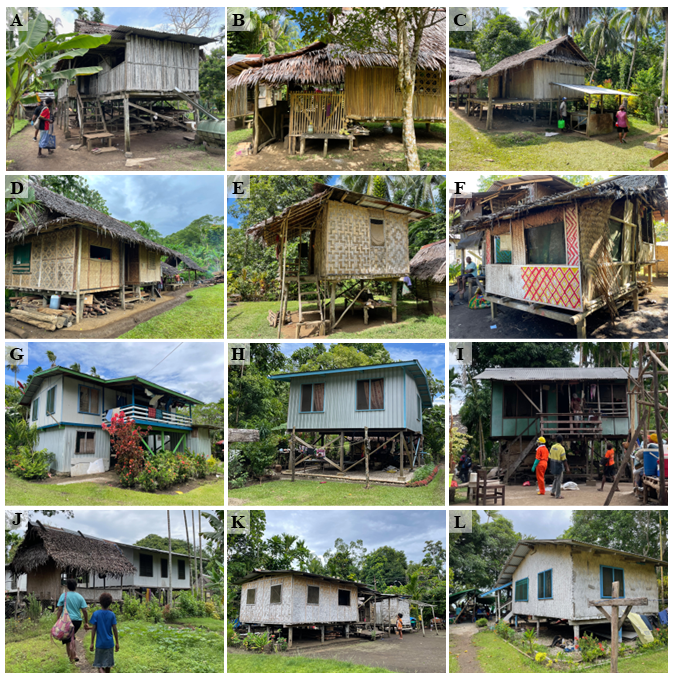


**Figure S1: Examples of diverse house types in study villages.** Houses were constructed from materials including sago palm (A, B, C), bamboo (D, E, F, K, L), metal sheet (G, H, J) and plywood (I). Outdoor sheltered areas targeted for spraying included walls on verandas (A, B, E, G, I), attached lean-tos (C) and separate gazebos (D, J).

**Figure S2: Correlation between deltamethrin and clothianidin.** The slope of the linear correlation between Deltamethrin and Clothianidin was 8.056, reflecting the mixture ratio of the two insecticides in the formulated product (1:8). Samples within the green square (63 %) were within the target application range.

**Figure S3: Comparison of the insecticide application rate between all study houses.** The numbers on the x-axis are the study household ID numbers and the surface materials (in parentheses). The horizontal lines indicate the target dose (red, continuous), and the +/- 50% range (dashed, grey). Data sorted by mean, from left to right.


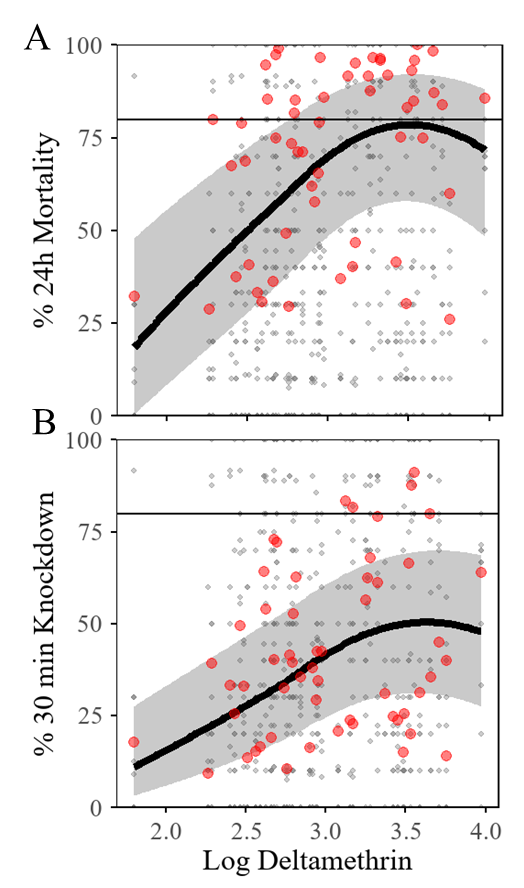


**Figure S4: Relationship between Deltamethrin concentration and mosquito mortality and knockdown.** Panel A: 24h mortality/Deltamethrin; Panel B: 30 min knock down/Deltamethrin. The red circles are the averages for all bioassays conducted on a surface with a particular deltamethrin concentration. The gray dots are the raw data for each individual assay.

## **Model Specifications**

*R codes for generalized mixed effects models*

**Model for M24h:** KM24h_Model <- glmer(cbind(Dead.24.h, nexposed - Dead.24.h) ~ daysfollowup + loc_bin + pos_cat + wallmaterial + logdeltamethrin + (1 | hhid/surface_ID) + (1 | ObsID), family = binomial, data = “Study_Data”, control = glmerControl(optimizer = "bobyqa", optCtrl = list(maxfun = 1e5))

**Model for KD30m:** KD30m_Model <- glmer(cbind(KD.30.min, nexposed - KD.30.min) ~ daysfollowup + loc_bin + pos_cat + wallmaterial + logdeltamethrin + (1 | hhid/surface_ID) + (1 | ObsID), family = binomial, weights = nexposed, data = “Study_Data”, control = glmerControl(optimizer = "bobyqa", optCtrl = list(maxfun = 1e5))

**Table S1: Model variable description**

| **Variable** | **Definition** | **Values** |
| --- | --- | --- |
| Dead.24.hr | Number of dead mosquitoes at 24h | 0 to 14 |
| KD.30.min | Number of knocked down mosquitoes at 30 minutes | 0 to 14 |
| daysfollowup | time of follow-up | 13 to 346 |
| loc_bin | Location of indoor and outdoor; | Indoor = 1  Outdoor =0 |
| pos_cat | Surface height category : low, medium, high, | High = 1  Medium = 2  Low = 3 |
| wallmaterial | Surface material category: bamboo sago; | Bamboo =1  Sago = 0 |
| deltamethrin | Concentration in mg per m^2^ | 6.04-53.1 |
| hhid: | household ID | 1016, 1018, 1065, 1090, 1092, 4055, 4057, 4122, 4203 |
| Surface_ID | Surface_ID within hhID | 1-6 |
| ObsID | Individual Observation ID | 1-573 |
| nexposed | Number of mosquitoes exposed | 3 to 14 |
